# The Notch and Wnt pathways regulate stemness and differentiation in human fallopian tube organoids

**DOI:** 10.1038/ncomms9989

**Published:** 2015-12-08

**Authors:** Mirjana Kessler, Karen Hoffmann, Volker Brinkmann, Oliver Thieck, Susan Jackisch, Benjamin Toelle, Hilmar Berger, Hans-Joachim Mollenkopf, Mandy Mangler, Jalid Sehouli, Christina Fotopoulou, Thomas F. Meyer

**Affiliations:** 1 Department of Molecular Biology, Max Planck Institute for Infection Biology, Charitéplatz 1, 10117 Berlin, Germany; 2 Core Facility Microscopy, Max Planck Institute for Infection Biology, Charitéplatz 1, 10117 Berlin, Germany; 3 Core Facility Microarray, Max Planck Institute for Infection Biology, Charitéplatz 1, 10117 Berlin, Germany; 4 Department of Gynecology, Charité University Medicine, Campus Mitte, Charitéplatz 1, 10117 Berlin, Germany; 5 Department of Gynecology, Charité University Medicine, Campus Virchow, Augustenburger Platz 1, 13353 Berlin, Germany

## Abstract

The epithelial lining of the fallopian tube is of critical importance for human reproduction and has been implicated as a site of origin of high-grade serous ovarian cancer. Here we report on the establishment of long-term, stable 3D organoid cultures from human fallopian tubes, indicative of the presence of adult stem cells. We show that single epithelial stem cells *in vitro* can give rise to differentiated organoids containing ciliated and secretory cells. Continuous growth and differentiation of organoids depend on both Wnt and Notch paracrine signalling. Microarray analysis reveals that inhibition of Notch signalling causes downregulation of stem cell-associated genes in parallel with decreased proliferation and increased numbers of ciliated cells and that organoids also respond to oestradiol and progesterone treatment in a physiological manner. Thus, our organoid model provides a much-needed basis for future investigations of signalling routes involved in health and disease of the fallopian tube.

## Abstract (web summary)

The mechanisms underlying fallopian tube epithelial renewal are unclear. Here, Kessler *et al.* isolate adult stem cells from the human fallopian tube epithelium and generate 3D organoids from these cells *in vitro* that have a similar architecture to that of the fallopian tube.

## Graphical abstract

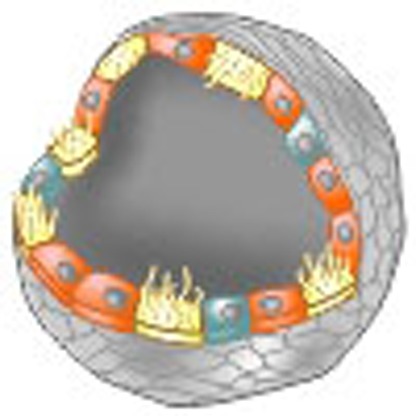

Adult stem cells of the human mucosa have been described in several tissues; however, the molecular mechanisms of renewal and differentiation remain obscure in many cases, including the fallopian tube, a central organ of human reproduction. It is lined by simple columnar epithelium containing secretory and ciliated cells, which produce tubular fluid and facilitate transport of gametes, respectively. In the fimbrium—the distal part that is in close contact with the ovary—this epithelium is organized into extensively branched mucosal folds. Since it is exposed to cyclical hormonal changes, mechanisms to ensure its long-term renewal and integrity are of critical importance. The presence of ‘stem cell-like' cells has previously been postulated based on sphere-forming capacity and differentiation *in vitro*^1^ and evidence for the presence of label-retaining cells in the distal fallopian tube^2^.

Here we demonstrate the existence of adult stem cells in the human fallopian tube epithelium, which gives rise to a monolayer of differentiated epithelial cells in a complex 3D organoid *in vitro*. They faithfully recapitulate the native tissue architecture, show robust growth and can be maintained long term in culture (>16 months so far). As in the intestinal tract, skin, liver and ovary^3^,^4^,^5^,^6^, stemness is maintained by active Wnt signalling and is centred on a subfamily of leucine-rich repeat-containing G protein-coupled receptors Lgr4, 5 and 6 (refs 7, 8, 9), which are themselves Wnt target genes, and have the capacity to strongly amplify Wnt signals, while the R-spondin family of proteins act as Lgr receptor agonists^10^. In congruence, the expansion capacity of organoids is modulated by Wnt3A and R-spondin-1 (RSPO1). Furthermore, Notch is responsible for the regulation of known stem cell factor genes. Our data reveal a significant overlap between Notch-dependent genes in the fallopian epithelium and the defined ‘stem cell signature' of the mouse intestine, suggesting the existence of a conserved pathway that regulates tissue renewal and directly controls cell fate specification and differentiation in the organoid by inhibiting cilliogenesis.

Therefore, this new organoid model closely mimics the normal physiology and anatomy of the human fallopian tube epithelium and provides a starting point for future investigations into the regulatory mechanisms involved in its cellular renewal and pathology.

## Results

### Adult fallopian tube organoids can be maintained long term

**Figure 1:**
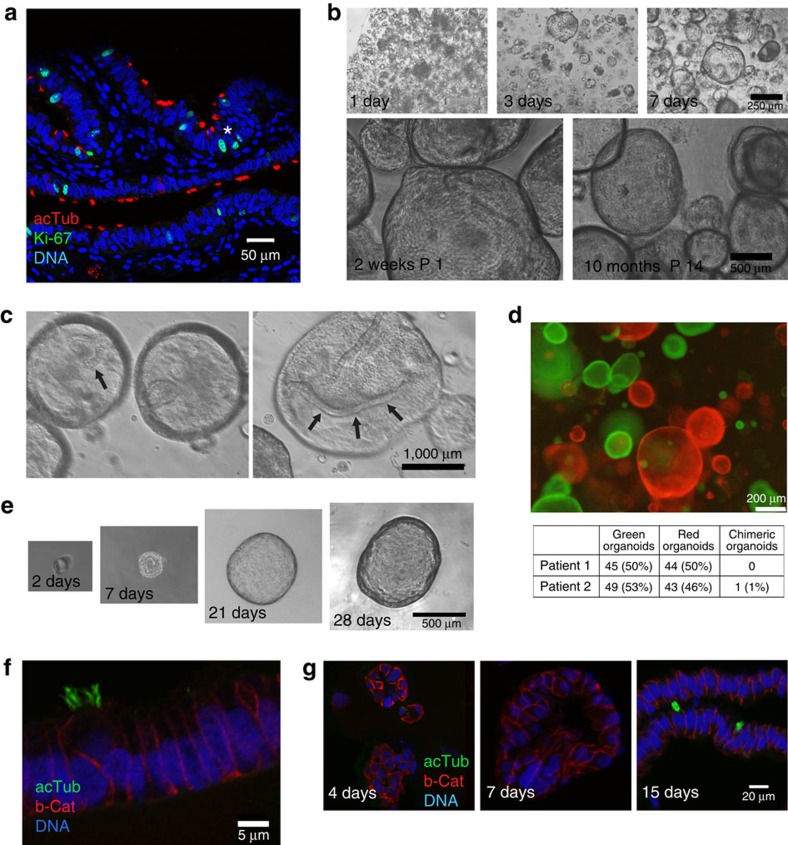
Single fallopian epithelial cells give rise to organoids that can be maintained long term. (**a**) Representative confocal image of fallopian tube tissue labelled for acetylated tubulin, a marker of ciliated cells (red), the proliferation marker Ki67 (green) and DNA (DRAQ5, blue). Ki67-positive nuclei are dispersed along epithelial folds, with occasional clustering (asterisk). (**b**) Phase contrast images of spheroid formation and growth. Small spheres are already visible 1 day after seeding and expand to reach a diameter of over 100 μm within 7 days. The same culture shows no apparent morphological differences after 2 weeks and after 10 months *in vitro*. (**c**) Phase contrast microscopy images of focal planes inside mature organoids showing the epithelial invaginations and folding (arrows), which are routinely present but not visible in most images focused on the organoid surface. (**d**) Representative image of organoids generated with independently labelled GFP and mCherry epithelial isolates showing almost exclusively single-colour organoids. Quantification of the organoids from two independent experiments and donors (table) reveals that the percentage of hybrids is on average below 1%. (**e**) Time course of a monoclonal organoid generated from a single EpCAM+ cell sorted and embedded in Matrigel in a 96-well format. Monoclonal organoid cultures were successfully generated from four different donor isolates, confirming the presence of stem cells in the fallopian tube epithelium. (**f**) Confocal microscopy image of a monoclonal organoid generated from a single fallopian tube stem cell after 2 months of 3D culture (P3), labelled for b-Cat (red) and tubulin, showing ciliated and non-ciliated cells. (**g**) Confocal images of early organoid cultures at different time points labelled for b-Cat (red) and ac tubulin (green), revealing that cilia develop after 2 weeks in 3D culture.

To obtain insight into the renewal activity of the human fallopian tube mucosa, we carried out immunofluorescence analysis of healthy samples from gynaecological tissue specimens. Ki-67-positive cells were distributed along the epithelial folds, without restriction to a particular region with occasional clustering (Fig. 1a, asterisk). The proportion was higher in the distal compared with the proximal region (5.0±0.3% versus 2.3±1.3%, respectively) in line with previous reports^11^.

To test whether these presumed stem cells can be maintained *in vitro* if given the appropriate paracrine environment, we isolated and seeded epithelial cells from patient samples in two-dimensional (2D) culture, followed by transfer to a three-dimensional (3D) Matrigel matrix, supplemented with a cocktail of growth factors to modulate Wnt, Notch, epidermal growth factor (EGF), fibroblast growth factor (FGF)-10 and transforming growth factor (TGF)-β signalling. Small round clusters of cells were visible after 24 h (Fig. 1b) and by 3 days post seeding developed into rapidly expanding spheres of a round, cystic phenotype, reaching up to 2.5 mm in diameter within 2 weeks. Each Matrigel drop seeded with 20,000 cells yielded an average of ∼700 spheroids. The appearance of folds, invaginations of the epithelium, as a hallmark of mature organoid culture is detected during the second week of 3D growth (Fig. 1c, arrows). Overall growth rates during long-term culture remained constant, with passaging at a 1:3 ratio required every 2–3 weeks (Fig. 1b). The doubling time of cells in organoid cultures, quantified based on the number of progeny from a defined number of seeded sells over 2 weeks of cultivation, was 3.5–5 days (Supplementary Fig. 1a), and Hoechst 33342 labelling to visualize proliferation revealed several mitotic cleavage planes within a small region of the organoid surface (Supplementary Fig. 1b), suggestive of rapid growth. Between passages individual organoids greatly increased in diameter (Supplementary Fig. 1c). Live cell imaging of fragmented pieces immediately after passage 13 (∼10 months) showed that they re-organized within 4–6 h, followed by rapid invagination of epithelial folds and spherical expansion (Supplementary Movie 1). Overall, this method has yielded expandable, stable organoid cultures in all healthy tissue samples from 52 donors tested so far, with only minor variations in sphere formation capacity and expansion rate between donors or between distal and proximal tubal regions (Supplementary Fig. 1d).

Freshly seeded epithelial isolates followed over 72 h using live cell imaging demonstrated that spheres do not form through the aggregation of cells but rather through the expansion of a subpopulation of individual cells (asterisks, Supplementary Movie 2). To investigate whether organoid formation is a monoclonal process, we labelled epithelial cells with either GFP- or mCherry-expressing lentiviruses, and seeded them in Matrigel at equal ratio. The resulting organoids were almost exclusively either red or green (Fig. 1d), with less than 1% dual-colour organoids (from non-clonal origin). We also performed clonogenic assays by seeding FACS-isolated epithelial cell adhesion molecule (EpCAM)+ cells at a ratio of one cell per 10 μl Matrigel drop in a 96-well format. Monoclonal organoids developed with a frequency of 0.5–1% (Fig. 1e), could be expanded long term (>3 months) and consisted of both secretory and ciliated cells, demonstrating the bipotency of the initiating progenitor cell (Fig. 1f). These stem cells are likely to be non-ciliated, since cilia were not seen in developing organoids until 2 weeks after initial cell seeding (Fig. 1g), despite the presence of ciliated cells in the initial cell isolate (Supplementary Fig. 1e). Hence, we conclude that organoid formation is vastly monoclonal and is driven by bipotent EpCAM+ stem cells of the fallopian tube, which maintain long-term potency in our system.

### Organoids faithfully recapitulate the mucosal fold architecture

**Figure 2:**
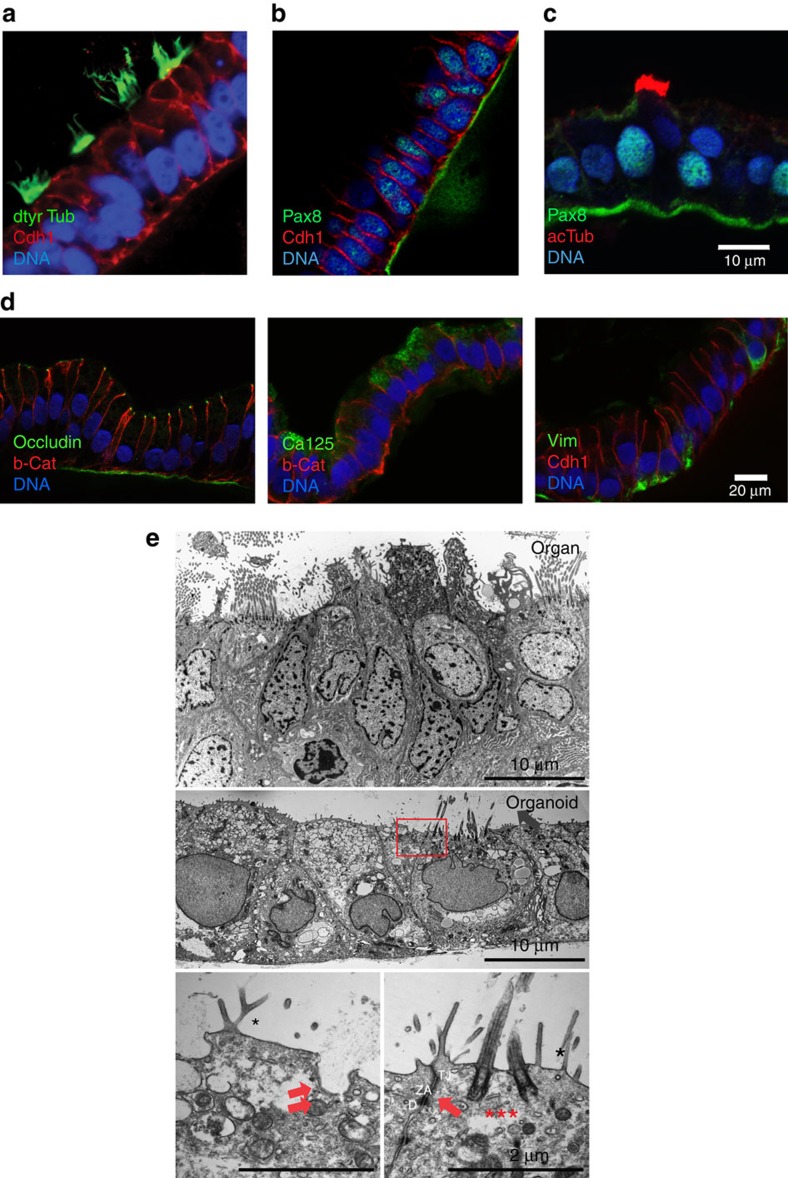
Organoids match the *in vivo* epithelium in structure and distribution of markers. Confocal images of organoid sections show that epithelium contains both ciliated cells positive for detyrosinated tubulin (green (**a**)) and secretory cells positive for PAX8 (green (**b**)) confirming differentiation. Terminally differentiated ciliated cells are PAX8-negative (**c**). (**d**) Epithelial cells form tight junctions visible by labelling for occludin (green, left). Organoids also contain occasional domains of epithelial cells ubiquitously expressing CA125 (green, middle) as well as basolateral vimentin (green, right). (**e**) Electron transmission microscopy images of the fallopian tube epithelium (upper panel) and organoid monolayer after 2 months *in vitro* (middle panel), showing high levels of similarity. Monolayer of the columnar epithelium with two cell types (ciliated and secretory), with clear apicobasal polarization closing off the empty lumen. Higher magnifications of the middle panel (square) reveal completely formed junctional complexes, which ensure separation of the apical from basolateral side (arrow, lower panel, right). The apical surface is covered by abundant microvilli (*) and fully developed cilia (***). Ruffling of the apical membrane on the non-ciliated cells (left panel) is highly suggestive of active release and secretion into the lumen (double arrow).

Detailed characterization of the organoid epithelium using confocal microscopy revealed highly polarized columnar cells, exhibiting precisely aligned nuclei, a restricted location of the main epithelial adhesion molecule E-Cadherin (Cdh-1) to the lateral membrane, and an orientation of the apical pole to the luminal side (Fig. 2a,b). Mature organoids contained Pax8-positive secretory cells as well as Pax8-negative, acetylated tubulin-positive ciliated cells. Occasional Pax8-positive ciliated cells (Fig. 2c), which were also observed in tissue sections (Supplementary Fig. 2a), suggest that loss of Pax8 represents a late event during differentiation to a ciliated phenotype, in line with our observation that both cell types derive from bipotent unciliated stem cells.

We further observed subapical localization of occludin in a nodal pattern, indicative of functional tight junctions (Fig. 2d, left). In addition, organoids contained clusters of cells expressing high levels of Ca125 protein (MUC16, Fig. 2d, centre), very similar to those observed in the tissue (Supplementary Fig. 2b). As previously shown for epithelial cells of the fallopian tube^12^, organoids also exhibited basolateral accumulation of vimentin (Fig. 2d, right). Transmission electron microscopy analysis (Fig. 2e) confirmed the high degree of overall similarity to fallopian tube tissue, including fully developed junctions between the cells consisting of desmosomes, zonula adherens and tight junctions (arrow, right panel), as well as fully assembled cilia (asterisks, right panel) and active secretion (double arrow, left panel), all of which indicate mature functional differentiation. Organoids also recapitulate the higher-order epithelial tissue architecture, as evidenced by frequent folding of epithelial sheets initially observed in phase-contrast images, is also prominent in confocal images of organoid sections, resembling the extensive folds seen in the authentic tissue (Supplementary Fig. 2b).

**Figure 3:**
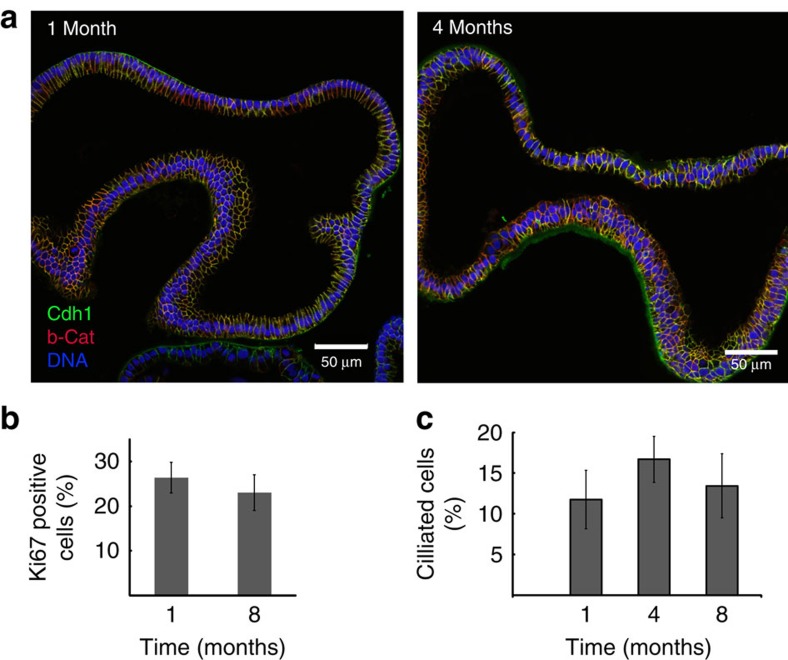
Organoid morphology and phenotype remains stable during long-term culture. (**a**) Comparative confocal analysis of the organoids after 1 and 4 months *in vitro* reveals stability of the phenotype, with unchanged morphology and staining pattern of the adhesion markers. (**b**) Dynamics of organoid growth remains stable over time as determined by quantification of the relative number of proliferative Ki-67-positive cells in confocal sections at 1- and 8-month culture. (**c**) The proportion of ciliated cells also remains unchanged as determined by quantification of ciliated cells. Error bars represent±s.d. from a minimum of five independent fields of view.

Comparative analysis of phenotypes at 1 and 4 months of culture showed long-term stability with no apparent changes in the epithelial structure and polarity over time, as organoids undergo a new cycle of growth after each passage (Fig. 3a). In agreement with the stable time window between passages, the percentage of actively proliferating cells also remained constant over time (Fig. 3b) as did the number of ciliated cells (Fig. 3c). We conclude that, in contrast to other organoid models^13^, the presence of Wnt3A and RSPO1 in the organoid culture medium maintains a subpopulation of stem cells long term, while at the same time allowing for full differentiation of the complete fallopian tube phenotypes.

### Active Wnt signalling preserves stemness in fallopian organoids

**Figure 4:**
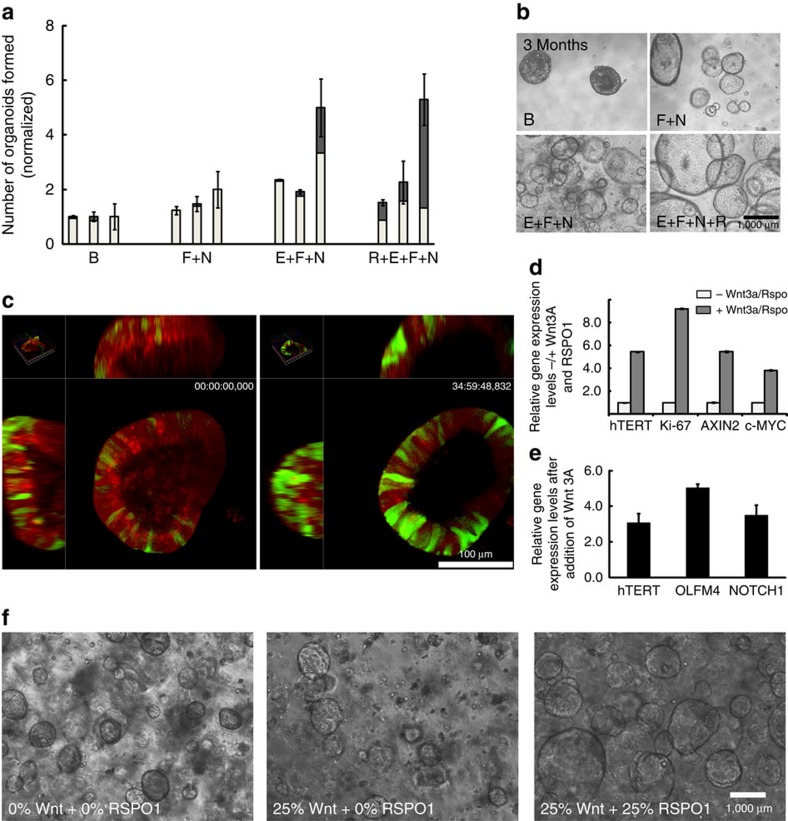
Active Wnt signalling is required for expression of stemness factors in the organoids and supports their growth. (**a**) Formation efficiency of the organoids determined by quantification in four different media with three independent donors (basic (B), FGF10 (F), noggin (N), EGF (E), RSPO1 (R)). Addition of EGF to the medium almost doubles sphere formation. Addition of RSPO1 strongly increases the size of the organoids, with a much higher number growing to above 300 μm diameter (dark grey fractions). Data are normalized to control conditions (B) ± cumulative s.d. of big and small organoids in duplicate wells. (**b**) During long-term expansion only organoids growing in complete medium maintain stable growth as seen in phase contrast images at 3 months in culture. (**c**) Active Wnt signalling is detected in the cells of the growing organoids by the TCF4-GFP reporter (green), which was introduced by lentiviral transduction. All cells containing reporter are labelled red (mCherry). Images are representative stills from a live-cell imaging experiment (Supplementary Movie 3). (**d**) qPCR analysis shows that addition of RSPO1 and Wnt3A to the medium for 7 days leads to a strong increase in the expression of *hTERT* and the proliferation marker *Ki67* relative to GAPDH (ΔΔ*C*_t_)^2^. Increased expression of the Wnt targets *AXIN2* and *cMYC* confirms pathway activation. Data are representative of two independent donor samples, and are represented as mean±s.d. of technical replicates. (**e**) Addition of 25% Wnt3A conditioned medium to 25% RSPO1 conditioned medium increases expression of Olfactomedin4 and Notch1, compared with control organoids grown in RSPO1 conditioned medium alone, as shown using qPCR. Data are representative of two independent patient samples and represented as mean±s.d. of technical replicates. (**f**) Phase contrast images of organoids 7 days post seeding in media supplemented with different Wnt pathway ligands. Pictures are representative of three independent patient samples. Notably, addition of Wnt3A on its own failed to improve sphere formation and growth, while RSPO1 greatly increased both number and size of spheres.

A requirement for active paracrine signalling to preserve functional stem cells has previously been described for gastrointestinal organoid models^13^. On the basis of the presumptive conservation of core renewal mechanisms, fallopian tube organoids were supplemented with EGF, FGF10 and factors that modulate paracrine Wnt signalling (Wnt3a and RSPO1), suppress TGF-β receptor kinase (ALK4/5) and BMP signalling (Noggin) and inhibit anoikis (ROCK inhibitor). Organoids did form in basic medium but their number doubled in the presence of EGF, while addition of RSPO1 increased their size (Fig. 4a). EGF and Wnt activation was crucial for maintenance of stable growth over time, as seen in comparative images from cultures maintained for 3 months with different supplements (Fig. 4b).

Although organoids could be initiated without TGF-β RK inhibitor (Supplementary Fig. 3a), they exhibited slowdown of expansion and finally growth arrest by four to six passages (3–4 months). In the presence of TGF-β RK inhibitor, on the other hand, quasi-indefinite expansion (>1 year) was seen routinely.

To demonstrate conclusively that Wnt signalling is active in fallopian tube organoids, we introduced the 7 × Tcf-eGFP reporter by lentiviral transduction, which contains an oligomerized TCF4-binding-site sequence, ensuring that expression is limited to cells in which this classical Wnt transcriptional response is initiated. Confocal live cell imaging over 40 h showed abundant green fluorescent protein (GFP)-positive cells (Fig. 4c), confirming activation of the canonical Wnt pathway. Notably, we detected several GFP-positive cells that were actively dividing (Supplementary Movie 3). In line with this, we independently confirmed transmission of the Wnt signal to the nuclei of early organoids with the lentiviral reporter T-cell factor (TCF)/lymphoid enhancer factor (LEF) DsRed (Supplementary Fig. 3b). To examine the importance of exogenous Wnt pathway stimulation, we used quantitative PCR (qPCR) to assess the effects of Wnt3A and RSPO1 on the expression of *hTERT*, a direct target of Wnt signalling^14^ reported to be highly expressed in intestinal stem cells^15^. Both Wnt ligands triggered strong upregulation of *hTERT* (Fig. 4d) and increased proliferation (Ki67), suggesting that this pathway is involved in maintaining fallopian tube stem cells. In addition, the Wnt target genes *Axin2* and *cMYC* were upregulated.

Next, we investigated whether there is interdependency between Wnt3A and RSPO1, and how this contributes to sphere formation and regulation of stemness. Wnt3A is the ligand of the canonical Wnt signalling pathway, which interacts with the LRP6/Frizzled receptor complex^16^. RSPO1 potentiates the cellular response to Wnts through a unique mode of action that involves binding to members of the LGR subfamily (LGR4, 5 and 6), without direct interaction with the Wnt receptor LRP6 (refs 6, 17, 18). To examine this interdependency, we measured expression of several stem cell genes using qPCR in the presence of RSPO1 alone or in combination with Wnt3A. Indeed, in the presence of RSPO1, Wnt3A addition strongly increased expression levels of *hTERT*, *Olfactomedin4* and *Notch1* (Fig. 4e). Without RSPO1, sphere formation capacity over 7 days was also very low even in the presence of Wnt3A (Fig. 4f). These data are consistent with other models showing that RSPO1 is an amplifier of Wnt3A signalling^10^. Thus, we conclude that RSPO1 plays a crucial role in formation and stem cell maintenance of fallopian tube organoids. Furthermore, induction of Olfactomedin4, a highly specific marker of the intestinal stem cell compartment^19^, might indicate a common organizing principle in these different niches. Likewise, Notch1 induction by Wnt ligands indicates interactions between the Wnt and Notch signalling pathways in the fallopian tube organoids and the concerted action of these two morphogens in stemness regulation.

### Notch signalling sustains expression of stem cell genes

**Figure 5:**
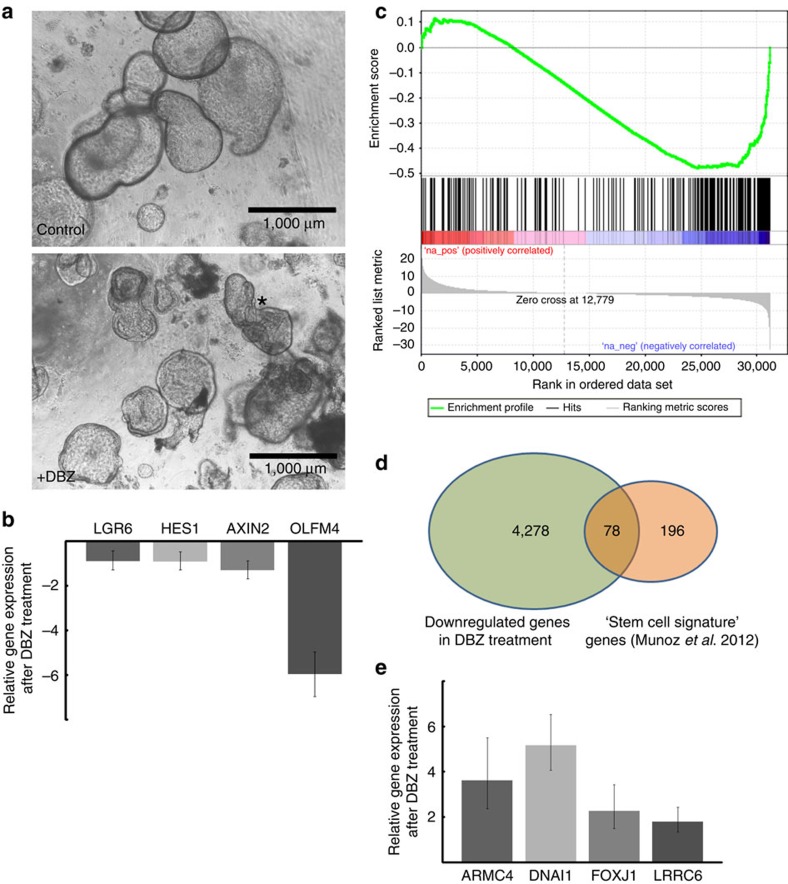
Inhibition of Notch signalling leads to downregulation of stemness-related genes. (**a**) Inhibition of the Notch pathway by addition of DBZ (1 μM; lower panel) changes the differentiation pattern and structure of the organoids, leading to distinct changes in morphology, evident by increased folding (asterisk). (**b**) qPCR validation of selected candidate genes that were found to be downregulated in the microarray. The stemness marker *Olfactomedin4*, Wnt signalling components *AXIN2* and *LGR6*, as well as the Notch target gene *HES1* were all confirmed to be downregulated in independent patient samples. Data represent mean±s.d. of three independent donors. (**c**) Gene Set Enrichment Analysis of the regulated genes after DBZ treatment identified by microarray, compared with a set of stem cell signature genes from mouse intestine. The correlation plot reveals a significant enrichment in the set of downregulated genes (negative *t*-score). (**d**) Venn diagram showing that 78 of the 274 ‘stem cell signature genes' were also found to be significantly downregulated in the fallopian tube organoids on inhibition of Notch. (**e**) qPCR validation of selected candidate genes functionally related to cillia found to be upregulated in the microarray. *ARMC4*, *DNAI1*, *FOXJ1* and *LRRC* were found to be consistently upregulated in four independent biological replicates. Data are presented as mean±s.d.

As the Notch signalling pathway is known to play a pivotal role in regulation of the adult intestinal stem cell niche^20^, we investigated whether growth of organoids and cell fate determination towards secretory or ciliated phenotypes is affected by inhibition of Notch by adding the γ-secretase inhibitor DBZ. Phase contrast imaging indicated increased folding within the organoids after 7 days of treatment (Fig. 5a, asterisk), and microarray analysis on the Agilent platform (44 k array) showed that DBZ caused a strong shift in the global gene expression pattern, affecting nearly 4,000 genes (cutoff at >1.5-fold regulation; see data deposited under the accession number GSE60919). Among the downregulated genes we identified several associated with the maintenance of stemness (*AXIN2*, *LGR6* and *Olfactomedin4*). This effect was validated using qPCR in samples from three independent patients (Fig. 5b). Effectiveness of DBZ treatment was confirmed by downregulation of the Notch signalling target *HES1*.

The observed downregulation of LGR6, the orphan transmembrane receptor belonging to the Wnt pathway, provides further evidence of the interplay between Wnt and Notch in the regulation of organoid growth and differentiation. LGR6 appears to be a good candidate for mediating the effects of RSPO1, based on the high sequence conservation and functional homology with LGR5 (refs 6, 18), which is not expressed in either the fallopian tube tissue or the organoids, as indicated by the microarray data. Immunofluorescence labelling revealed a randomly distributed population of LGR6-positive cells in both tissue and organ sections (Supplementary Fig. 4). In the organoids these cells were often, but not exclusively, found in regions enriched in Ki67 (Supplementary Fig. 4a, asterisks).

On the basis of the hypothesis that the Notch pathway plays a role in the maintenance of the fallopian stem cell niche, we used systematic Gene Set Enrichment Analysis (GSEA) to test whether the downregulated genes are part of a larger molecular programme related to stemness. For comparison, we used the human homologues of a set of 274 previously described adult mouse intestinal crypt ‘stem cell signature' genes^21^. On the basis of the similarity of the paracrine signalling pathways involved, we wanted to examine whether the underlying conserved molecular mechanisms could be identified. GSEA revealed that the set of downregulated genes in DBZ-treated organoids is significantly enriched (GSEA permutation test, *P*<0.001) for the intestinal ‘stem cell signature' genes (Fig. 5c). Of the 274 genes, 78 were significantly downregulated (log ratio error, *P*<0.05) after Notch inhibition (Fig. 5d) including *Eph4A*, *RNF43*, *SMO* and the frizzled receptors *FZD2* and *FZD7* (Supplementary Table 1), all of which are known to play important roles in preservation of stemness^22^,^23^,^24^. Thus, conserved molecular mechanisms regulating adult stem cell niches are active in the fallopian tube, and expression of stemness-related genes in the epithelium is supported by active Notch signalling.

### Regulation of differentiated cells in the organoid

**Figure 6:**
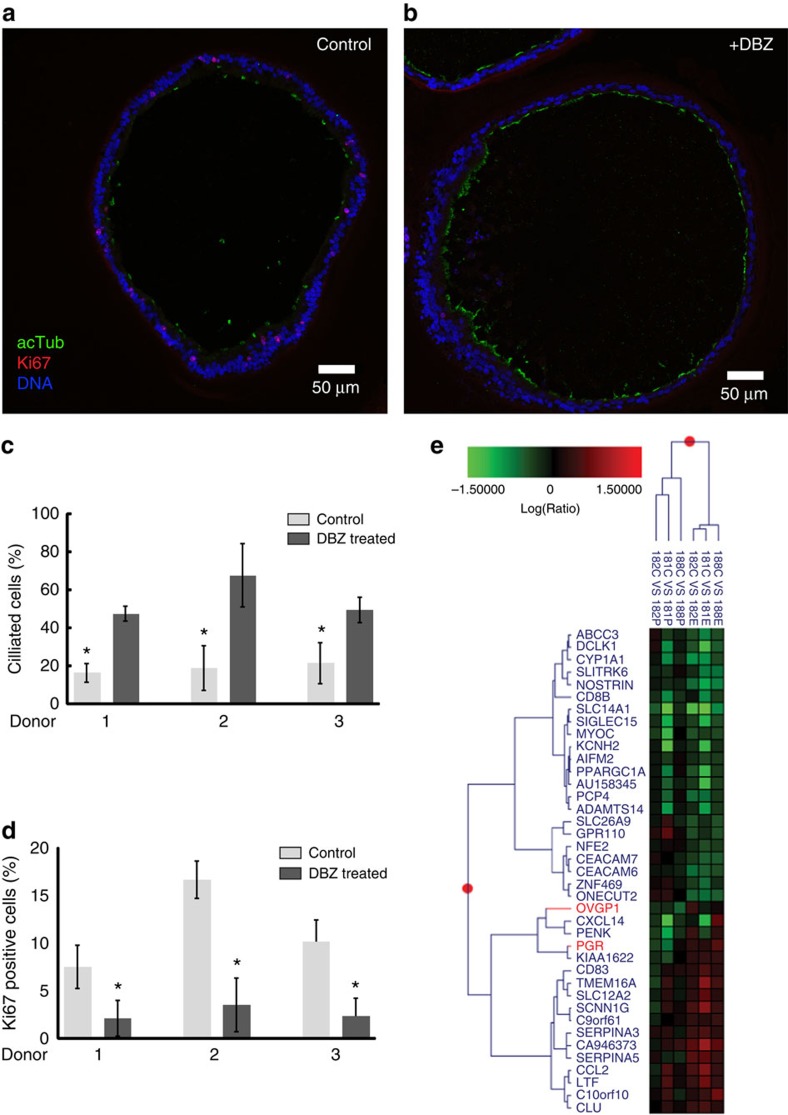
Notch signalling is required for maintaining a secretory phenotype. Representative confocal images of control (**a**) and DBZ-treated (1 μM) organoids (**b**), showing an increase in the number of ciliated cells positive for acetylated tubulin (green), as well as a decrease in the number of proliferating cells positive for Ki67 (red) when Notch is inhibited. (**c**,**d**) Quantification of ciliated and actively proliferating cells in control versus DBZ-treated organoid sections from three different donors. Error bars represent±s.d. from a minimum of seven independent fields of view. (**e**) Organoids respond to oestradiol (500 pmol l^−1^) and progesterone (50 ng ml^−1^) stimulation as determined by gene expression profiling by microarray analysis of three independent donors. The colour matrix of the heat map depicts the log10(Ratio) of individual unstimulated (C) versus hormone-stimulated (estradiol, E or progesterone, P) comparisons while the hierarchical structures are illustrated as dendrograms at gene and patient levels.

Downregulation of stemness-promoting genes should be accompanied by reduced proliferation as well as increased differentiation. To obtain a systematic, unbiased overview, we used the online GOrilla platform^25^,^26^ to perform gene ontology enrichment analysis of the list of genes regulated by Notch that were identified with microarray. The programme identified cilia and accessory microtubuli as highly enriched primary cellular components (Supplementary Fig. 5), suggesting that the Notch pathway promotes a secretory cell phenotype and that inhibition triggers differentiation into ciliated cells. To validate the microarray data, the expression levels of several genes indispensable for ciliogenesis and/or cilia function were analysed using qPCR: *ARMC4*, *DNAI1*, *FOXJ1* and *LRRC6* (refs 27, 28, 29, 30). Organoids from four different donors revealed a strong induction of all four genes after DBZ treatment for 7 days (Fig. 5e). Confocal analysis of organoids from three further patients confirmed that DBZ treatment reduced the proportion of Ki67-positive cells while simultaneously increasing the proportion of ciliated cells more than twofold (Fig. 6a–d). Therefore, renewal of the tubal epithelium depends on the Notch pathway, and differentiation into different phenotypes can be modulated by signal strength. This might have significant implications for understanding tubal epithelium pathology, as most models of tubal carcinogenesis postulate ‘secretory cell outgrowth'^31^ as the initial step in transformation.

Since the fallopian tube is responsive to a hormonally active environment that plays a role in the aetiology of disease, we tested the responsiveness of organoids to stimulation with oestradiol (500 pmol l^−1^) or progesterone (50 ng ml^−1^) for 2 weeks. Global gene expression pattern analysis by microarray identified more than 1,500, and 450 genes regulated in response to oestradiol and progesterone treatments, respectively (Fig. 6e). Several of these are known to be inversely regulated during different phases of the menstrual cycle, such as progesterone receptor. We also observed a strong oestradiol-induced upregulation of oviductal glycoprotein 1 (*OVGP1*) thought to play an important role during fertilization. Analysis of genes significantly regulated across three donors reveals clustering for both types of treatment (oestradiol or progesterone) and direction of regulation. Thus, our model should be valuable for studies on the role of hormones in the regulation of the fallopian tube epithelium.

## Discussion

Here we report the establishment of human fallopian tube organoids that faithfully recapitulate the phenotype of *in vivo* tissue and can be stably expanded for more than a year without apparent changes in phenotype, essentially demonstrating the existence of fallopian tube stem cells. These are single bi-potent stem cells that give rise to whole organoids through long-term proliferation and differentiation into secretory and ciliated cells. Successful cultivation depends on supplementation with appropriate growth factors that support paracrine signalling pathways, in particular Wnt and Notch, to preserve the stem cell niche. The absence of these supplements is a likely reason for previous failures in expanding fallopian tube cells *in vitro* and their incomplete polarization, resulting in abundant multilayering and the absence of a central cavity^12^.

The effects of Notch inhibition strongly support a central role of the Wnt and Notch pathways in the regulation of stemness. The overlap between Notch-dependent genes in the human fallopian tube and the stem cell signature network of the mouse intestine^21^ points to similar regulatory mechanisms in these two niches. LGR5-positive intestinal stem cells are known to exhibit high telomerase activity *in vitro*^15^, which rapidly decreases in transit amplifying and differentiating cells. Thus, it is likely that the increase in expression of *hTERT*, *Ki67*, *Olfactomedin4* and *Notch1* that we observed when active Wnt signalling is maintained by RSPO1 and Wnt3A reflects an increase in the number of stem cells.

The requirement for RSPO1 further indicates a decisive role of its receptors for epithelial tissue renewal in the fallopian tube. Our microarray data reveal an insignificant expression of LGR5, the main receptor for RSPO1 in gastrointestinal tissues. In line with this, a recent report also showed only very limited expression in the human fimbrium and none in the adult mouse fallopian tube^6^. In contrast, we detected strong expression of its homologue *LGR6* in tissue sections as well as organoids, suggesting that it could be a functional RSPO1 receptor, and potentially also a stem cell marker in the fallopian tube.

Our findings have considerable implications for the understanding of fallopian epithelial renewal *in vivo* and its role in disease development. While cellular transformation of the ovarian surface epithelium is still a possibility^32^, it is the epithelium of the fimbrium that is now widely considered as the most frequent tissue of origin of high-grade serous ovarian cancer, while intestine and uterus are likely to be additional tissues contributing to the aetiology of this peritoneal malignancy^33^,^34^. While the exact contribution of each of these organs to this highly complex disease is yet to be determined, there is clearly a need for a better understanding of the biology of fallopian tube epithelium. In this context, our organoid model from patient material, exhibiting the polarized epithelium that readily responds to hormonal stimulation opens up exciting new possibilities for studying the aetiology of ovarian cancer. Deregulation of Notch signalling has been identified as one of the most frequent and significant changes in ovarian cancer patients^35^. Thus, our finding that Notch is an important regulator of stemness and differentiation in the healthy fallopian tube provides important insight for the cellular mechanism that maintains homeostasis. The presence of PAX8+/acTub+ ciliated cells in both tissue and organoids strongly suggests that the secretory phenotype preceeds development of cilia and could prove to be crucial for understanding early transformation events in the fallopian tube, which have been defined as ‘outgrowths of secretory cells'^31^. The described fallopian tube organoid model thus promises to be a valuable tool for future investigations on the biology and pathology of this relatively inaccessible organ, particularly with regard to tumour biology, as well as reproductive medicine. The observed long-term stability and expansion potential of the human fallopian tube tissue provides substantial advantages over existing primary cell models enabling numerous downstream experimental applications, such as the generation of mutant organoids and the advancement of screening technology^36^.

## Methods

### Antibodies and chemicals

The following primary antibodies were used: mouse monoclonal anti-E-Cadherin (BD Biosciences, Cat. No. 610181, 1:200), rabbit polyclonal anti-Ki 67 (Cell Signaling, Cat. No. 9027, 1:100), rabbit polyclonal anti-β-Catenin (Sigma, Cat. No. C2206, 1:50), rabbit polyclonal anti-detyrosinated tubulin (Abcam, Cat. No. 48389, 1:100), rabbit polyclonal anti-Pax8 (Proteintech, Cat. No. 10336-1-AP, 1:50), mouse monoclonal anti-acetylated tubulin (Sigma, Cat. No. 6-11-B1, 1:100), rabbit polyclonal anti-LGR6 (Antibodiesonline, Cat. No. ABIN122588, 1:100), mouse monoclonal anti-Ca 125 (Calbiochem, Cat. No. Ca1004, 1:100), mouse monoclonal anti-occludin (Invitrogen, Cat. No. 33-1500, 1:100) and rabbit polyclonal anti-vimentin (Cell Signaling, Cat. No. 5741, 1:100). We further used the following: Draq5 (Thermofisher Scientific, Cat. No. 62251), γ-secretase Inhibitor XX (Calbiochem, Cat. No. CAS 209984-56-5), β-oestradiol (Sigma, Cat. No. E2758) and progesterone (Acros Organics, Cat. No. Cas 57-83-0).

### Epithelial progenitor isolation from fallopian tube samples

Human fallopian tube samples were provided by the Department of Gynecology, Charité University Hospital, Campus Virchow Clinic and Campus Charité, Berlin, Germany. Scientific usage of the samples for experimental purposes was approved by the Ethics Commission of the Charité, Berlin (EA1/002/07), and all subjects gave informed consent to their tissue being used in scientific research. Fragments were sourced from standard surgical procedures for benign gynecological disease. Only anatomically normal fallopian tubes were used. Tubes were transported and dissected within 2–3 h of removal.

Samples were washed with Dulbecco's PBS (DPBS), and excessive connective and vascular tissue was removed. Tubes were opened longitudinally to expose the mucosal folds, washed with DPBS and incubated with 0.5 mg ml^−1^ collagenase type I (Sigma) for 45 min at 37 °C to dissociate the epithelial cells from the extracellular matrix. The mucosal cells were then scraped off with a scalpel and collected in a Falcon tube. After centrifugation (3 min at 25 °C, 300*g*) the cell pellet was resuspended in ADF (Invitrogen), 12 mM HEPES, 1% GlutaMAX (both from Invitrogen), 5% FCS (Biochrom), supplemented with 10 ng ml^−1^ human EGF (Invitrogen), 9 μM ROCK inhibitor (Y-27632, Sigma) and 1% penicillin/streptomycin (Invitrogen), seeded in uncoated cell culture flasks and kept at 37 °C, 5% CO_2_ in a humidified incubator.

### Organoid culture

Once they reached 70% confluence, epithelial cells were detached with TrypLE Express (Gibco), centrifuged (7 min at 4 °C, 300*g*) and seeded in 50 μl Matrigel (Corning) in pre-warmed 24-well plates at a density of 25,000 cells per well. The Matrigel was solidified for 20 min at 37 °C and overlaid with 500 μl pre-warmed expansion medium (ADF, 25% conditioned human Wnt3A medium as described in ref. 37 and 25% conditioned human RSPO1 medium, as described in ref. 38, supplemented with 12 mM HEPES, 1% GlutaMAX, 2% B27, 1% N2, 10 ng ml^−1^ human EGF (all from Invitrogen), 100 ng ml^−1^ human noggin, 100 ng ml^−1^ human FGF10 (both from Peprotech), 1 mM nicotinamide, 9 μM ROCK inhibitor (Y-27632, both from Sigma) and 0.5 μM TGF-β R Kinase Inhibitor IV (SB431542, Calbiochem). Cultures were kept at 37 °C, 5% CO_2_ in a humidified incubator. A tissue fragment of ∼5 cm yields a confluent monolayer of epithelial cells containing ∼8 × 10^6^ cells, within 5 days post isolation. These cells can be efficiently transferred to 3D, and could potentially yield ∼400 wells seeded at 20,000 cells per Matrigel drop. With an average expansion rate of approximately ninefold between passages (Supplementary Fig. 1a), this would yield almost 10^8^ cells within 3 weeks of isolation.

Basic medium (Fig. 3a) contained ADF supplemented with 12 mM HEPES, 1% GlutaMAX, 2% B27, 1% N2, 1 mM nicotinamide and 0.5 μM TGF-β R Kinase Inhibitor IV.

For hormone stimulation experiments, organoids were treated for 2 weeks with beta-oestradiol (500 pmol l^−1^ l), progesterone (50 ng ml^−1^) or mock-treated (alcohol) in standard 3D growth medium described above. Subsequently, RNA was isolated and microarray was performed.

### FACS isolation of EpCAM+ cells and single-cell culture

Isolated epithelial cells at P0 were detached with TrypLE and labelled for 25 min on ice with fluorescein isothiocyanate-conjugated anti-human CD326 (EpCAM) antibody (Miltenyi Biotec, 130-080-301, 1:50) diluted in ADF medium supplemented with 1% N2, 2% B27, 1 mM nicotinamide, 1 mM acetylcysteine and 9 μM ROCK inhibitor, according to Jung *et al.*^39^ EpCAM-positive single cells were sorted by FACS with exclusion of dead cells using propidium iodide (see Supplementary Fig. 6). Sorted single cells were embedded in Matrigel in a 96-well format at a dilution of one cell per well and cultured as described above.

### Maintenance of long-term cultures

The medium was exchanged every 2–4 days, and spheroids were passaged every 14–21 days. For passaging, the Matrigel containing the spheroids was dissolved in 1 ml cold ADF. Mechanical fragmentation was achieved by vigorous pipetting (8–10 times) with a fire-polished glass Pasteur pipette in a 15-ml Falcon tube. Sheared spheroids were centrifuged for 5 min at 4 °C, 300*g*. The resulting pellet was resuspended in cold Matrigel and replated at the desired density.

### Lentiviral manipulation

Replication-deficient lentiviral particles were produced by transfection of 293T cells (ATCC CRL-11268) using 2 × HBS (50 mM HEPES, 280 mM NaCl and 1.5 mM Na_2_HPO_4_), 1 M CaCl_2_ and a mixture of the following vectors: packaging vector psPAX2 (Addgene plasmid 12260), envelope vector pMD2.G (Addgene plasmid 12259) and the 7TGC lentiviral plasmid (Wnt reporter system with GFP and mCherry expression, Addgene plasmid 24304), the pLVTHM-GFP or pLVTHM-mCherry plasmid (Addgene plasmid 12247) or the TCF/LEF BAT red (Addgene plasmid 20674). Two days after transfection, the supernatant containing the lentiviral particles was aspirated, filtered (0.45 μm) and concentrated with Lenti-X Concentrator (Clontech). The lentiviral pellet was dissolved in the ADF medium.

For transduction, fallopian tube epithelial cells were seeded at early passage (P0 or P1) in six-well plates and grown to 40–50% confluence. Then, 100 μl of the 10 × concentrated lentiviral particles carrying the respective plasmid were taken up in 1 ml ADF medium supplemented with 12 mM HEPES, 1% GlutaMAX , 10 ng ml^−1^ human EGF, 9 μM ROCK inhibitor and 8 μg ml^−1^ polybrene (Sigma). The cells were incubated with the 1 × lentivirus-containing medium (1 ml per well) overnight. When the cells reached ∼70% confluence, an organoid culture was prepared as described above.

### Immunohistochemistry

Organoids were released from Matrigel by washing with cold PBS, fixed in 3.7% paraformaldehyde for 1 h at room temperature, dehydrated in an ascending series of alcohols, followed by isopropanol and acetone (all steps 20 min at room temperature). Finally, the dehydrated organoids were paraffin-embedded and cut into 5-μm sections on a microtome. Immunofluorescent labelling was carried out as described previously^18^. In brief, sections were rehydrated, treated with antigen retrieval solution (Dako), blocked for 1 h in PBS with 1% BSA and 2% FCS, incubated for 1.5 h with primary antibodies diluted in blocking buffer, washed with PBS-T (PBS with 0.1% Tween 20), incubated with secondary antibodies for 1 h, washed with PBS-T and mounted in Mowiol (Sigma).

### Confocal microscopy and scanning electron microscopy

Fresh epithelial isolates were grown on coverslips and fixed with 2% paraformaldehyde/PBS. Cells were permeablized with 0.5% Triton X-100, blocked and stained with antibodies against acetylated tubulin (Sigma, 6-11-B1, 1:1,000) and PAX8 (Proteintech, 10336-1-AP, 1:50), followed by a secondary antibody coupled to Alexafluor 488 (Jakson Immuno, 715-545-150, 1:300) and to Cy3 (Dianova, 711-166-152, 1:300), respectively.

Confocal stacks were recorded using a Leica TCS SP5 confocal microscope and processed for 3D modelling with the Volocity 6.3 software package. A parallel sample was fixed with 2.5% glutaraldehyde, postfixed with osmiumtetroxide, contrasted using tannic acid and dehydrated in an ethanol series. After critical point drying, specimens were coated with 3-nm carbon/platinum and analysed using a LEO1550 field emission scanning electron microscope.

### Transmission electron microscopy

Human fallopian tube tissue as well as organoids derived thereof were fixed with 2.5% glutaraldehyde, postfixed with osmiumtetroxide, contrasted using tannic acid and uranyl acetate, dehydrated in an ethanol series and embedded in Poly/Bed 812. After polymerization, sections were cut at 70 nm using a diamond knife. Sections were contrasted with lead citrate and analysed in a LEO906E transmission electron microscope.

### Image analysis and cell counts

Image analysis was performed with an SP-8 confocal microscope (Leica). Numbers of ciliated and Ki67-positive cells were determined using wide angle (× 20) images covering ∼200–700 cells per image in ImageJ with the ‘cell counter' plugin. Seven fields of view were counted for control and DBZ-treated organoids, equivalent to a total of >2,000 cells per condition.

### RNA quantification and quality control

Total RNA was isolated with TRIzol (Life Technologies) according to the supplier's protocol, using glycogen as the carrier. Quality control and quantification of total RNA were assessed using an Agilent 2100 Bioanalyzer with an RNA Nano 6000 microfluidics kit (Agilent Technologies) and a NanoDrop 1000 UV-Vis spectrophotometer (Kisker).

### Microarray expression profiling and data analysis

Microarray experiments were performed as dual-colour hybridizations. To compensate for dye-specific effects and to ensure statistically relevant data, colour-swap, dye-reversal hybridizations were performed^40^. RNA labelling was performed with a two-colour Quick Amp Labeling Kit according to the supplier's recommendations (Agilent Technologies). In brief, mRNA was reverse-transcribed and amplified using an oligo-dT-T7 promoter primer and labelled with Cyanine 3-CTP or Cyanine 5-CTP. After precipitation, purification and quantification, 1.25 μg of each labelled cRNA was fragmented and hybridized to whole-human genome 4 × 44 K multipack microarrays (Design ID 014850) according to the manufacturer's protocol (Agilent Technologies). Scanning of microarrays was performed with 5 μm resolution using a G2565CA high-resolution laser microarray scanner (Agilent Technologies) with extended dynamic range (XDR). Microarray image data were analysed and extracted with the Image Analysis/Feature Extraction software G2567AA v. A.11.5.1.1 (Agilent Technologies), using default settings and the protocol GE2_1100_Jul11. The extracted MAGE-ML files were subsequently analysed with the Rosetta Resolver, Build 7.2.2 SP1.31 (Rosetta Biosoftware). Ratio profiles comprising single hybridizations were combined in an error-weighted manner to create ratio experiments. A 1.5-fold change expression cutoff for ratio experiments was applied together with anticorrelation of ratio profiles, rendering the microarray analysis highly significant (log ratio error, *P*<0.01), robust and reproducible.

For analysis of hormonal stimulation arrays, genes identified as either up- or downregulated for all three patients under at least one hormone condition with an assigned function were 2D-clustered without statistical cuts with the Agglomerative algorithm and the heuristic criterion Average link using the similarity measure Cosine correlation.

### Real-time PCR

Organoids were released from Matrigel with cold DPBS and pelleted by centrifugation (7 min at 4 °C, 300*g*), followed by RNA isolation using either the GeneJET RNA Purification Kit (Fermentas) or RNeasy Mini Kit (Qiagen) according to the manufacturer's protocol. Real-time PCR was performed using the Power SYBR Green RNA-to-CT 1-Step Kit (Life Technologies), on an ABI Prism 7900 H sequence detection system (Applied Biosystems). Reactions were performed in 25 μl containing 50–200 ng RNA, 10 μl SYBR Green mix, 0.16 μl RT mix and 0.2 μM per primer.

Programme: 30 min 48 °C; 10 min 95 °C; followed by 40 × cycles of 15 s 95 °C/60 s 60 °C. Primers: *glyceraldehyde-3-phosphate dehydrogenase* forward 5′-GGTATCGTGGAAGGACTCATGAC-3′, reverse 5′-ATGCCAGTGAGCTTCCCGTTCAG-3′; *OLFM4* forward 5′-ACTGTCCGAATTGACATCATGG-3′, reverse 5′-TTCTGAGCTTCCACCAAAACTC-3′; *AXIN2* forward 5′-CCTGCCACCAAGACCTACAT-3′, reverse 5′-CTTCATTCAAGGTGGGGAGA-3′; *HES1* forward 5′-TTCTGAGCTTCCACCAAAACTC-3′, reverse 5′-AGGCGCAATCCAATATGAAC-3′; hTERT forward 5′-CGGAAGAGTGTCTGGAGCAA-3′, reverse 5′-GGATGAAGCGGAGTCTGGA-3′; *Ki-67* forward 5′-AAGCCCTCCAGCTCCTAGTC-3′, reverse 5′-TCCGAAGCACCACTTCTTCT-3′, *LGR6* forward 5′-CCTTCCTCAGCTTCACCTTG-3′, reverse 5′-CTTCCAGGCCTTTCTCTGTG-3′; *Notch1* forward 5′-AAACAAGTGAAAGCATATGGGTTAGAT-3′, reverse 5′-CCTGAAACAAAGATTCATGATTGGT-3′, *ARMC4* forward 5′-AAATCCTCACCCAGACGTG-3′, reverse 5′-ATGTTGGTAATGGCAGCACA-3′, *DNAI1* forward 5′-GCGTTTTGTAGGCTCTCCAG-3′, reverse 5′-CCCATTCATCTGTGTGCCTTCT-3′, *LRRC6* forward 5′-ACCACCTACAGGCACCAGAC-3′, reverse 5′-TCCTGGGTGGTTTCACTTTC-3′, *FOXJ1* forward 5′-GGAGGGGACGTAAATCCCTA-3′, reverse 5′-GGTCCCAGTAGTTCCAGCAA-3′.

For each oligonucleotide pair and RNA sample, the reaction was performed in triplicate. The amplification plots obtained from the RT–PCR were analysed with the ABI Prism SDS Software package (version 2.2.2; Applied Biosystems). The expression levels were quantified applying the comparative *C*_t_ (threshold cycle) method and calculating ΔΔ*C*_t_. Relative expression levels of the target genes were normalized to the expression of glyceraldehyde-3-phosphate dehydrogenase in each individual sample.

Fold change was calculated as an average of ΔΔ*C*_t_ of independent biological replicates (2^−^ΔΔ*C*_t_), while s.d. was calculated as ΔΔ*C*_t_+*s* and ΔΔ*C*_t_–*s*, where *s* is the pooled s.d. of the Δ*C*_t_ and Δ*C*_t_ control values 
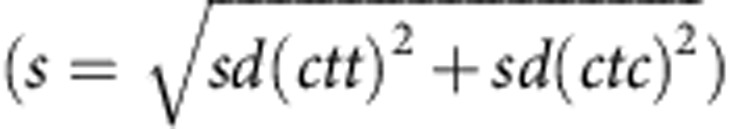
.

### Gene Set Enrichment Analysis

Overall, 274 unique human homologues of 384 mouse genes previously published as the stem cell signature genes of LGR5+ cells in intestinal crypts^21^ were identified using data from the Inparanoid data base^41^. GSEA^42^ for that list was performed on genes pre-ranked by gene expression-based *t*-score between DBZ-treated and non-treated organoids, using standard settings with 1,000 permutations.

### Gene ontology enrichment analysis

The list of all upregulated target genes (cutoff >1.5-fold) was uploaded to the GOrilla online platform, a tool for identifying and visualizing enriched GO terms in ranked lists of genes. An analysis was performed for the ontology term ‘Cellular Components'. Output is provided as a colour-coded diagram with all significantly enriched GEO terms.

## Additional information

**Accession codes**: The microarray data have been deposited in the National Center for Biotechnology Information Gene Expression Omnibus (GEO) under accession code GSE60919.

**How to cite this article**: Kessler, M. *et al.* The Notch and Wnt pathways regulate stemness and differentiation in human fallopian tube organoids. *Nat. Commun.* 6:8989 doi: 10.1038/ncomms9989 (2015).

## Supplementary Material

Supplementary Information Supplementary Figures 1-6, Supplementary Table 1 and Supplementary References.

Supplementary Movie 1 Live cell imaging of long term organoid culture (10 months). Live cell imaging of the growth of a long-term organoid culture over 72 h immediately after passage and seeding of fragments in fresh Matrigel. After initial rearrangements of the folds, the organoid continues expansion and spheric growth, following a pulse-like growth pattern.

Supplementary Movie 2 Initiation of organoid culture from single cell suspension. Live cell imaging of the formation of spheroids from a fallopian tube epithelial cell suspension seeded in Matrigel over 72 h. Notably, fast growing spheroids arise from tiny cell clusters (asterisks) and not from large aggregates.

Supplementary Movie 3 Active Wnt signaling in organoids. Confocal live cell imaging of an organoid transfected with TCF4/GFP reporter during 72 h showing dynamic Wnt activation. Notable are actively dividing GFP positive cells indicative of proliferative activity driven by the Wnt pathway
