# Supplementary material for: The Notch and Wnt pathways regulate stemness and differentiation in human fallopian tube organoids
Source: Nat Commun. 2015 Dec 8;6:8989. doi: 10.1038/ncomms9989 (PMC4686873; doi:10.1038/ncomms9989)
Supplement: Supplementary Information — Supplementary Figures 1-6, Supplementary Table 1 and Supplementary References. [file ncomms9989-s1.pdf]

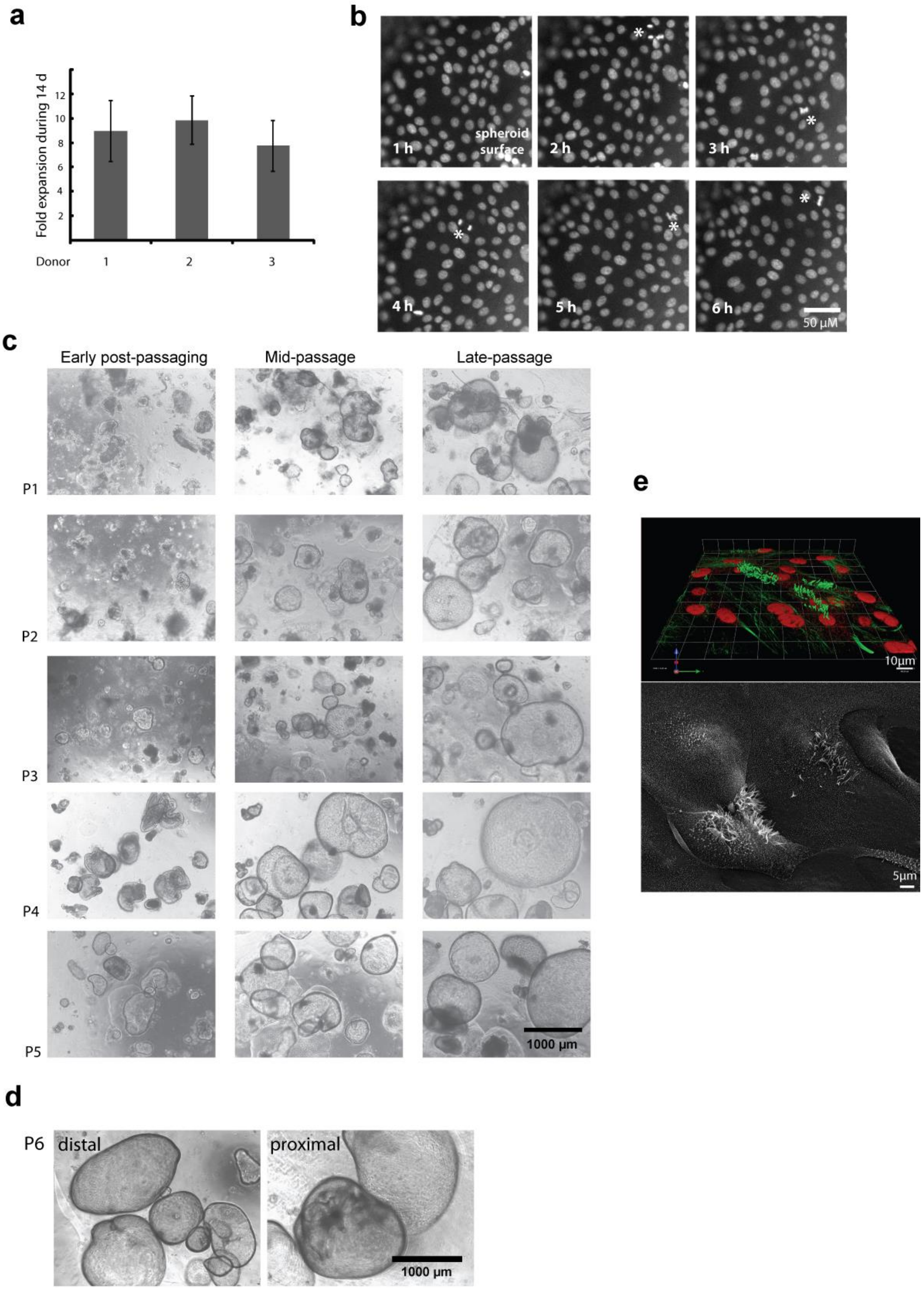

a
Fold expansion during 14 d
12
10
8
6
4
2
Donor
1
2
3
b
spheroid surface
1 h
2 h
3 h
4 h
5 h
6 h
50 μM
c
Early post-passaging
Mid-passage
Late-passage
P1
P2
P3
P4
P5
1000 μm
d
P6
distal
proximal
1000 μm
e
10μm
5μm

Supplementary Fig. 1 Fallopian tube organoids show robust and stable growth in long-term culture

a) Cell proliferation during two weeks of culture, determined for organoids from 3 different donors in passage 0 (donor 1), passage 2 (donor 2) and passage 4 (donor 3). Error bars represent S.D. of five technical replicates for each condition. **b**) Representative images of the surface of a growing organoid labeled with Hoechst 33342 for 6 h. Several mitotic planes (asterisks) are clearly visible in actively dividing cells. **c**) Overview of growth in a representative long-term 3D culture over the course of five passages, showing increasing size of individual organoids during a single passage. **d**) Phase contrast images of organoids in passage 6 (3 months) generated from the distal and proximal parts of the tube, reveal a stable phenotype from both regions **e**) Immunofluorescence labeling of epithelial isolates from fallopian tube tissue shows PAX8 positive cells as well as ciliated cells (upper panel). Cilia are also visualized by scanning electron microscopy (lower panel).

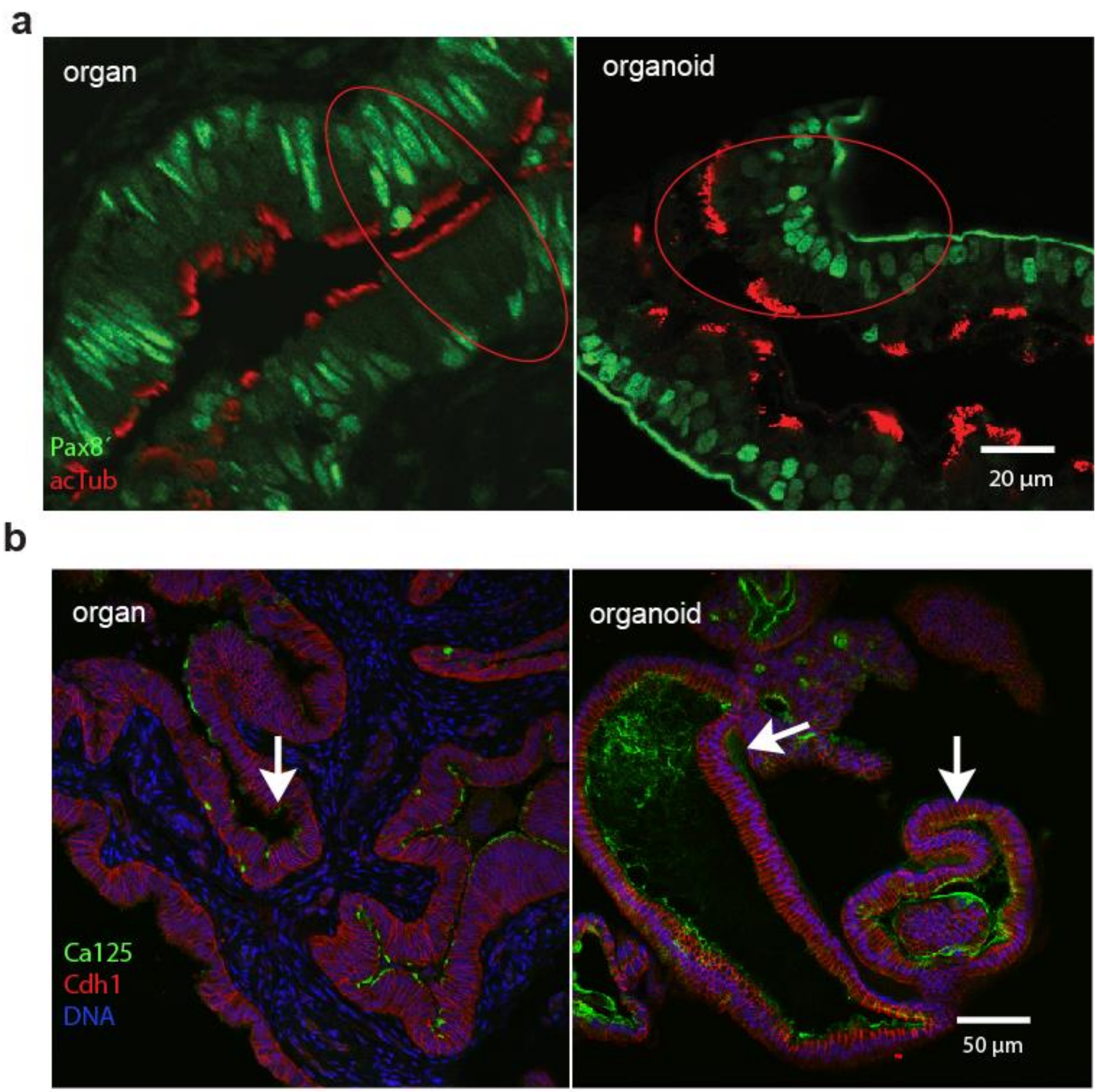

Supplementary Fig. 2 Organoids express functional and differentiation markers of native fallopian tube tissue

a) Confocal images of organoid (85 days *in vitro*) and tissue sections with PAX8 (green) and ac tubulin (red) reveal the presence of both PAX8 negative ciliated cells as well as PAX8/ac tubulin double positive cells (red circles). **b**) Organoids show a similar pattern of Ca125 expression (left, green) to the one observed in tissue (right). Confocal images of organoids also confirm extensive epithelial folding (arrows).

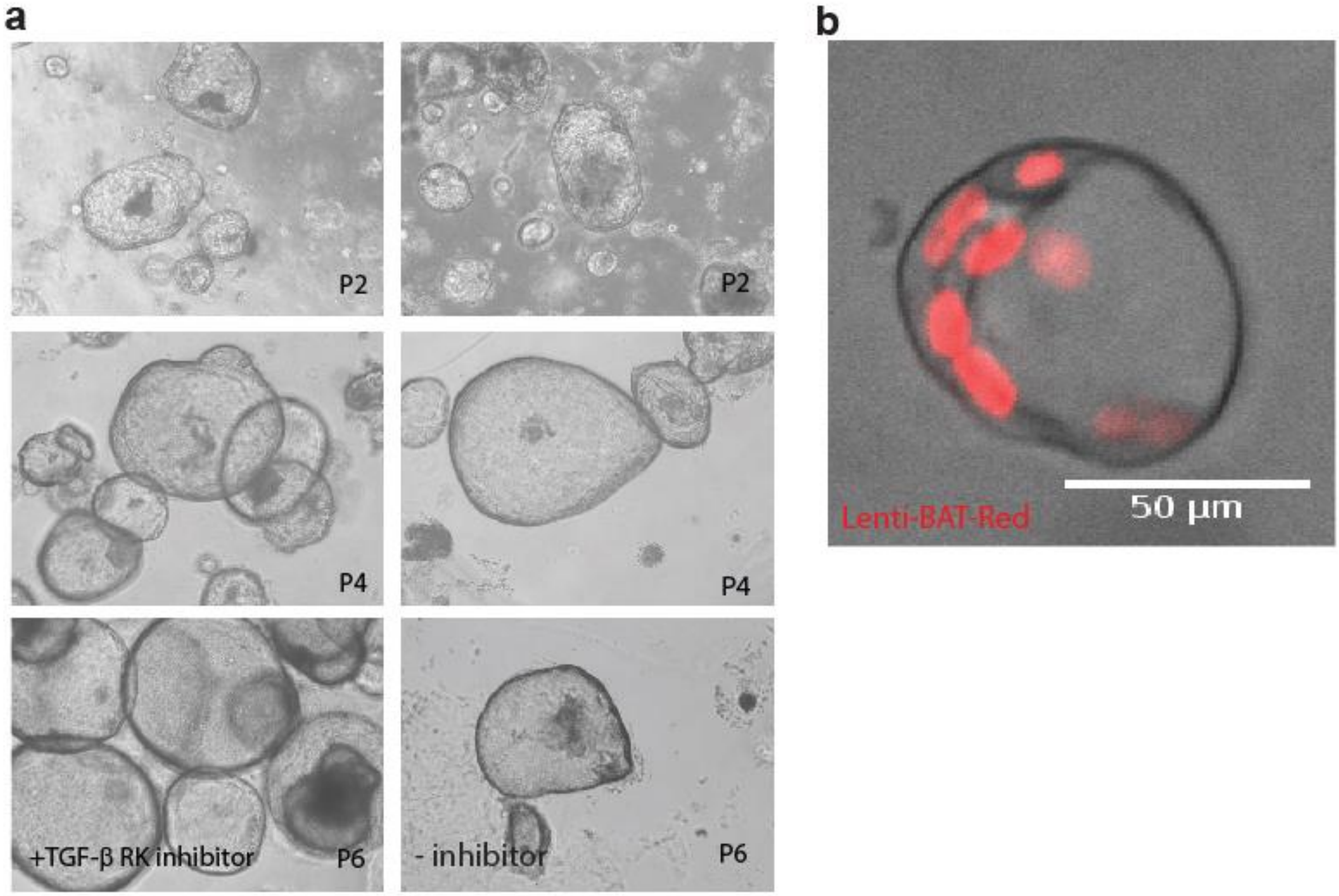

Supplementary Fig. 3 Suppression of TGF-β signaling is required for long-term organoid cultivation

a) Phase contrast images of organoid cultures expanded with or without TGF-β receptor kinase (RK) inhibitor. Expansion of cultures beyond passage 6 was only possible with continuous TGF-β RK inhibitor supplementation. **b**) Epifluorescence image of the TCF/LEF (Lenti-Bat–Red reporter) in an early organoid, confirming Wnt activation.

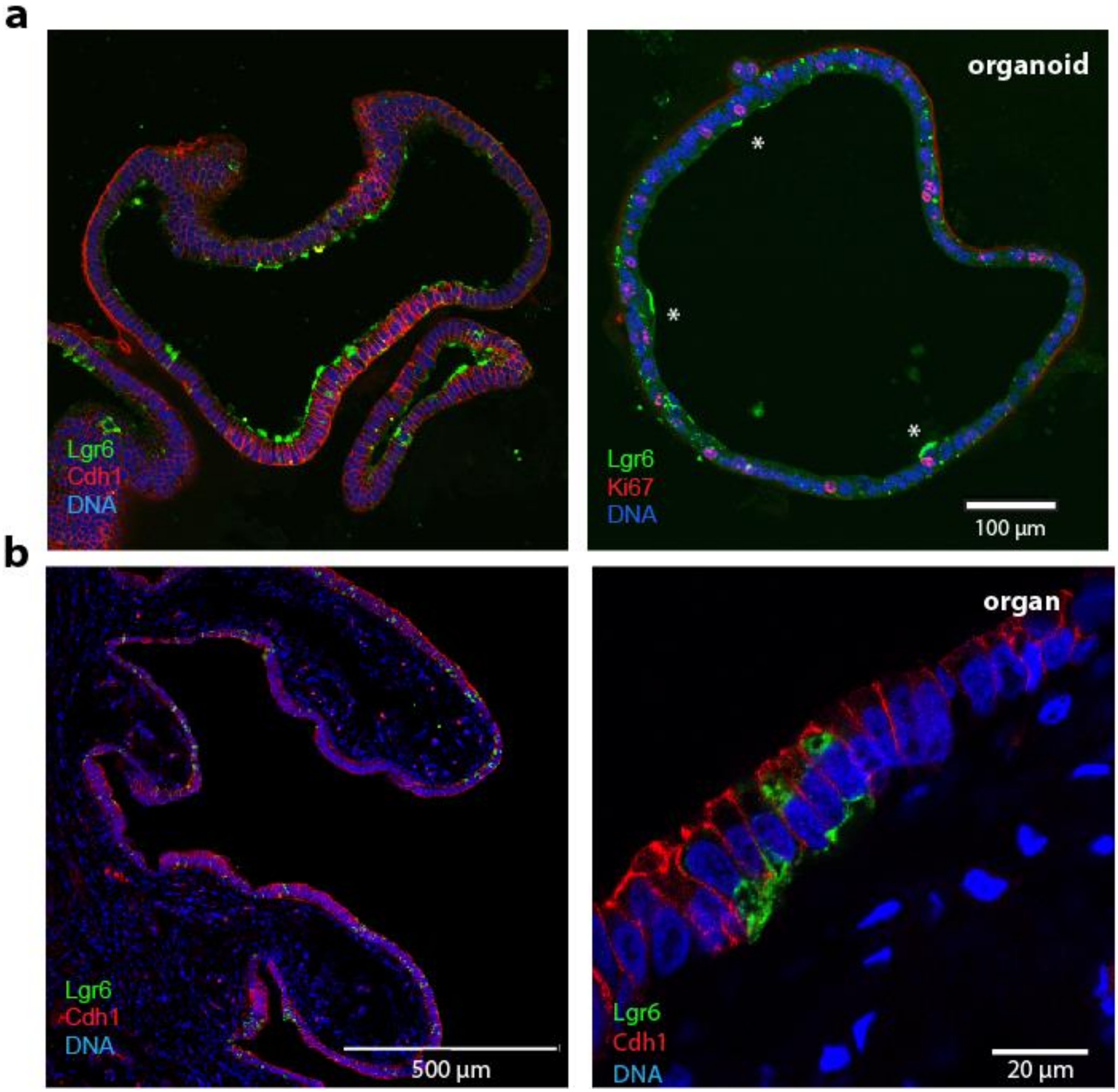

Supplementary Fig. 4 Organoids express Lgr 6
Representative confocal images showing the distribution of Lgr6 positive cells (green) in growing organoids (**a**) and in fallopian tube sections (**b**).

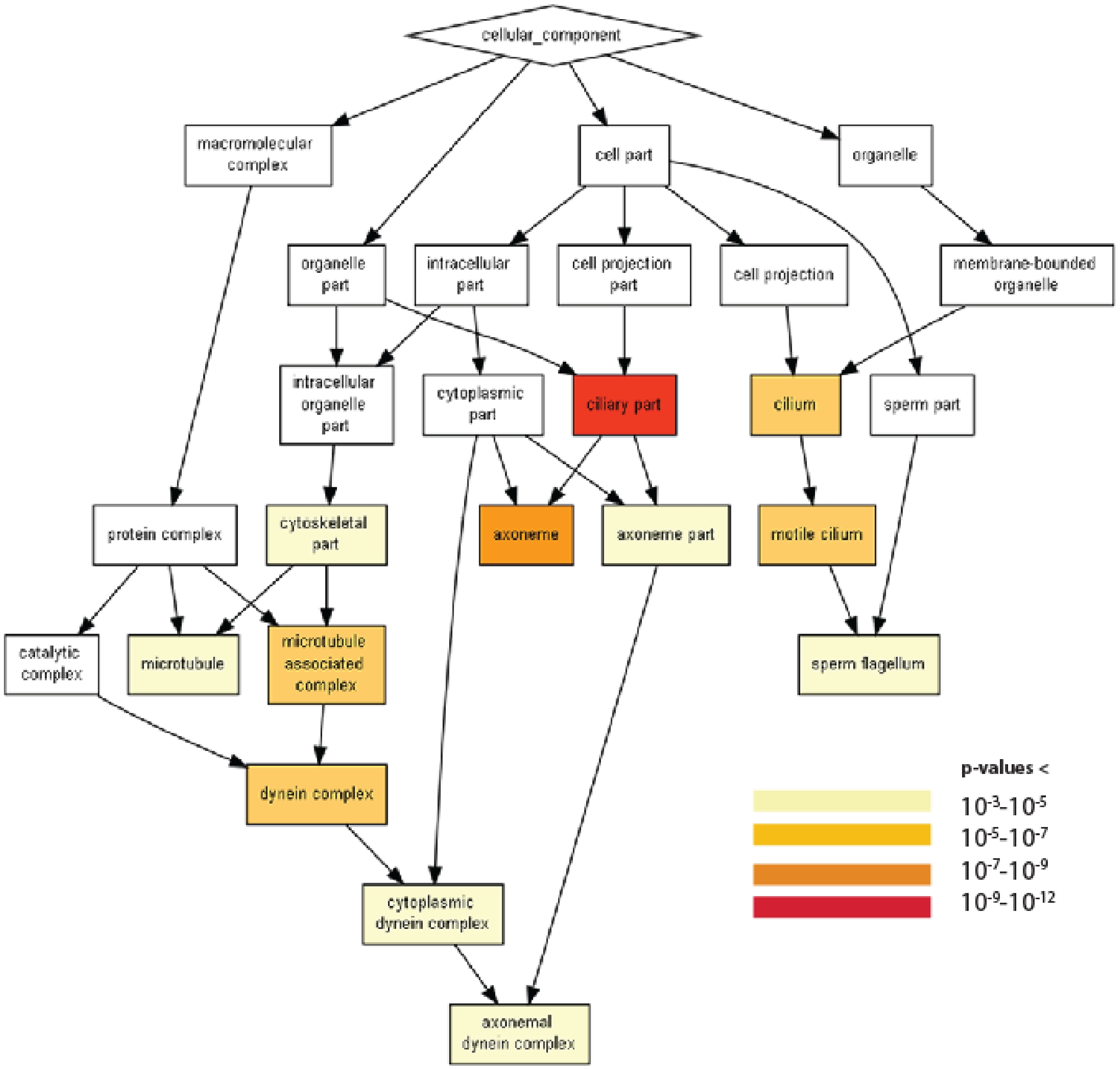

Supplementary Fig. 5 Cluster of genes involved in ciliogenesis is upregulated in organoids upon Notch inhibition

Diagram illustrating enrichment of cilia-related genes in the list of upregulated targets (from the Notch inhibition microarray), obtained using the GOrilla platform. Color is indicative of p value, and therefore of scale of enrichment for a particular node, calculated by minimum hypergeometric score (mHG) method [1].

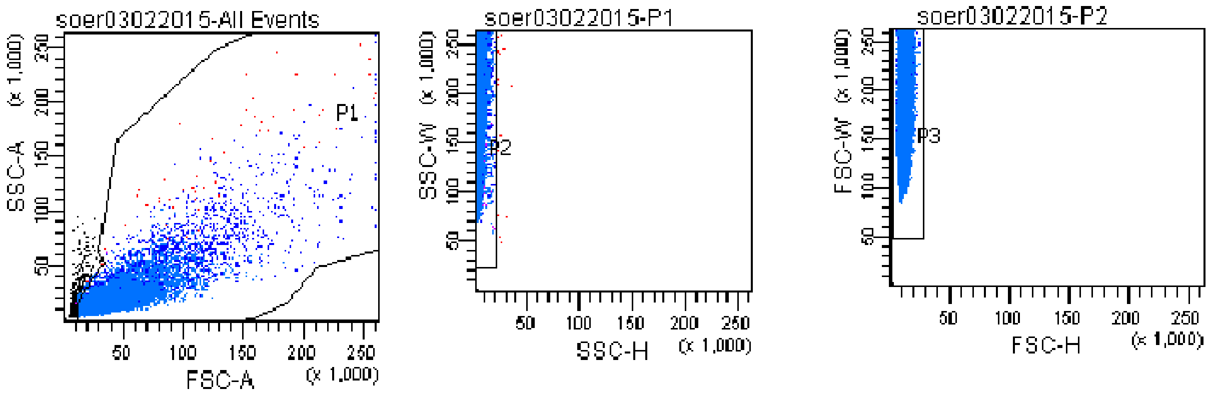

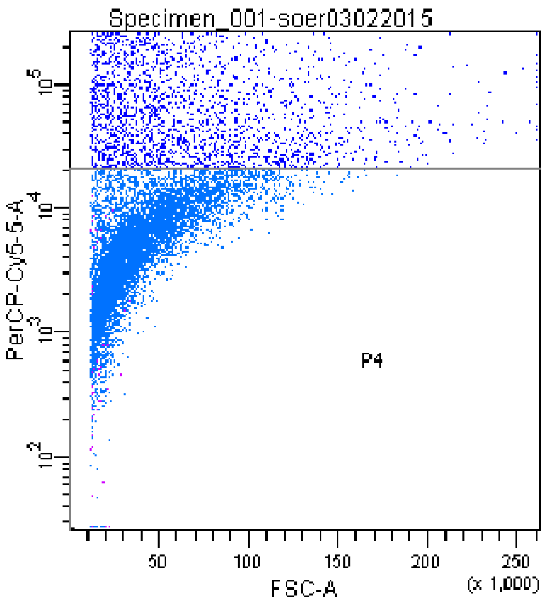

Tube: soer03022015

Population	#Events	%Parent	%Total
All Events	12,727	####	100.0
P1	11,310	88.9	88.9
P2	11,244	99.4	88.3
P3	11,244	100.0	88.3
P4	8,320	74.0	65.4
P5	8,166	98.1	64.2

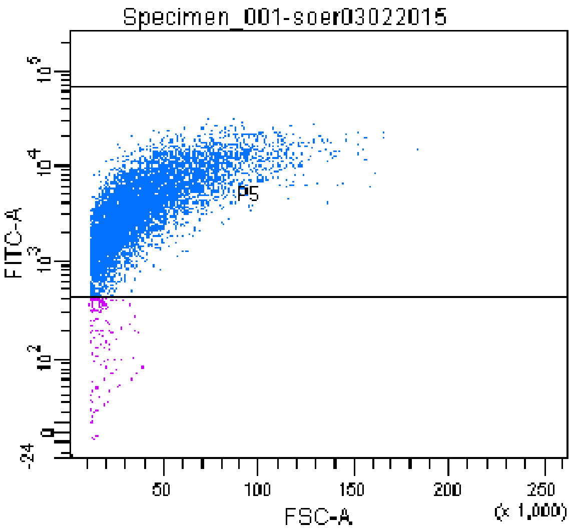

Supplementary Fig. 6 Fluorescence-activated cell sorting of single EpCAM+ cells
Example of FACS sorting results for primary isolates from fallopian tube tissue showing high purity of EpCAM+ epithelial cells (>98%) which were then used in dilution for clonality assay in a 96-well format.

Supplementary Table 1 Stem cell signature genes significantly downregulated in fallopian tube organoids upon DBZ treatment

Symbol	Gene name
ACPL2	NA
ARL4C	ADP-ribosylation factor-like 4C
ATM	ATM serine/threonine kinase
AXIN2	axin 2
BCL2	B-cell CLL/lymphoma 2
CA12	carbonic anhydrase XII
CD320	CD320 molecule
CDCA7	cell division cycle associated 7
CDK6	cyclin-dependent kinase 6
CITED4	Cbp/p300-interacting transactivator, with Glu/Asp-rich carboxy-terminal domain, 4
CKAP2	cytoskeleton associated protein 2
CNN3	calponin 3, acidic
DACH1	dachshund family transcription factor 1
DCTD	dCMP deaminase
EFNA4	ephrin-A4
EHF	ets homologous factor
EPHA4	EPH receptor A4
EVL	Enah/Vasp-like
FARP1	FERM, RhoGEF (ARHGEF) and pleckstrin domain protein 1 (chondrocyte-derived)
FSTL1	follistatin-like 1
FZD2	frizzled class receptor 2
FZD7	frizzled class receptor 7
GAS6	growth arrest-specific 6
GINS1	GINS complex subunit 1 (Psf1 homolog)
GREB1	growth regulation by estrogen in breast cancer 1
IL17RD	interleukin 17 receptor D
ILF3	interleukin enhancer binding factor 3, 90kDa
IMPDH2	IMP (inosine 5'-monophosphate) dehydrogenase 2
IRF2BP2	interferon regulatory factor 2 binding protein 2
KCNE3	potassium channel, voltage gated subfamily E regulatory beta subunit 3
KCNQ1	potassium channel, voltage gated KQT-like subfamily Q, member 1
KIAA0922	KIAA0922
KLHL23	kelch-like family member 23
KLHL24	kelch-like family member 24
KRT23	keratin 23, type I
LRIG1	leucine-rich repeats and immunoglobulin-like domains 1
MAN2A2	mannosidase, alpha, class 2A, member 2
MCC	mutated in colorectal cancers
MFGE8	milk fat globule-EGF factor 8 protein
MPP3	membrane protein, palmitoylated 3 (MAGUK p55 subfamily member 3)
MPZL1	myelin protein zero-like 1
MYC	v-myc avian myelocytomatosis viral oncogene homolog
MYO1B	myosin IB
NAV1	neuron navigator 1
OLFM4	olfactomedin 4
PAICS	phosphoribosylaminoimidazole carboxylase, phosphoribosylaminoimidazole succinocarboxamide synthetase
PCDH8	protocadherin 8
PCK2	phosphoenolpyruvate carboxykinase 2 (mitochondrial)
PHLDB2	pleckstrin homology-like domain, family B, member 2
PLXNB1	plexin B1
POGK	pogo transposable element with KRAB domain
PRELP	proline/arginine-rich end leucine-rich repeat protein

PRKACB	protein kinase, cAMP-dependent, catalytic, beta
PRKD3	protein kinase D3
RASL11B	RAS-like, family 11, member B
RHOBTB3	Rho-related BTB domain containing 3
RNF43	ring finger protein 43
SEMA7A	semaphorin 7A, GPI membrane anchor (John Milton Hagen blood group)
SLC19A2	solute carrier family 19 (thiamine transporter), member 2
SLC23A3	solute carrier family 23, member 3
SMO	smoothened, frizzled class receptor
SORCS2	sortilin-related VPS10 domain containing receptor 2
SOX4	SRY (sex determining region Y)-box 4
SOX9	SRY (sex determining region Y)-box 9
SP5	Sp5 transcription factor
TEAD2	TEA domain family member 2
TGIF2	TGFB-induced factor homeobox 2
TIMELESS	timeless circadian clock
TLR2	toll-like receptor 2
TMEM132A	transmembrane protein 132A
TNS3	tensin 3
TRIM24	tripartite motif containing 24
TUBB2B	tubulin, beta 2B class IIb
ZBTB38	zinc finger and BTB domain containing 38
ZNF462	zinc finger protein 462
ZNF618	zinc finger protein 618
ZNRF3	zinc and ring finger 3
ZRANB3	zinc finger, RAN-binding domain containing 3

Supplementary References

1. Eden, E., Navon, R., Steinfeld, I., Lipson, D. & Yakhini, Z. GOrilla: a tool for discovery and visualization of enriched GO terms in ranked gene lists. *BMC Bioinformatics* **10**, 48 (2009).
